# Spindle cell sarcoma: a case report of diagnostic and therapeutic quandary in a low resource setting

**DOI:** 10.1093/jscr/rjab612

**Published:** 2022-01-21

**Authors:** Sagar Panthi, Sajana Poudel, Nimesh Khanal, Siddhartha Bhandari, Seema Adhikari, Pradeep Khatiwada, Bharosha Bhattarai, Susmit Sharma, Sandeep Khanal, Suresh P Shah

**Keywords:** Plastic Surgery

## Abstract

Sarcomas can present differently in different parts of the body and showcase varied histopathological features and tend to recur locally and metastasize to distant sites. We discuss a case of a 37-year-old male with local recurrence of spindle cell sarcoma of the paraspinal muscles of size 20 × 20 cm^2^ with overlying ulceration and discharge with possible pulmonary metastasis. The mass was evaluated using magnetic resonance imaging/computed tomography and the histology was confirmed by biopsy. Wide surgical resection of the mass was done and the patient was referred to another center for radiotherapy and further treatment. The large size of the sarcoma and the possible pulmonary metastasis poses a risk of significant morbidity and mortality in this patient. This case showcases the scenario of many patients in developing countries where the patients are lost to follow-up due to various reasons and present later with grave consequences.

## INTRODUCTION

Sarcomas are a rare group of malignant tumors arising from the mesenchymal tissue, which make <1% of all adult malignancies [1–2]. World Health Organization classifies the group of soft-tissue neoplasm into >100 different histological subtypes based on the presumptive tissue of origin and their architectural pattern [2]. A huge group comprises undifferentiated soft-tissue sarcomas [3]. Sarcomas usually present as a painless mass and rarely present with distant metastasis especially in the lungs [4].

Spindle cell sarcoma is one of the rare varieties of undifferentiated soft-tissue sarcomas [5]. Due to its rarity, only a few cases have been described in medical literature [5]. Here, we report a case of a 37-year-old man with spindle cell sarcoma of the paraspinal muscles, which recurred locally after surgery.

## CASE REPORT

We present a case of a 37-year-old male presenting to the surgery out-patient department with a mass in the left upper back, which had grown into present size in 1 year. He had a history of a mass in the same location (5 × 4 cm^2^), which was excised as liposarcoma in another hospital and referred to a cancer hospital for further evaluation. On revision biopsy, spindle cell sarcoma was diagnosed and the patient was advised follow-up in the same hospital for radiotherapy of the tumor bed. Due to his ignorance and financial problems, he did not follow-up and ignored the growing mass and presented to our hospital only after having pain and ulceration over the mass.

**Figure 1:**
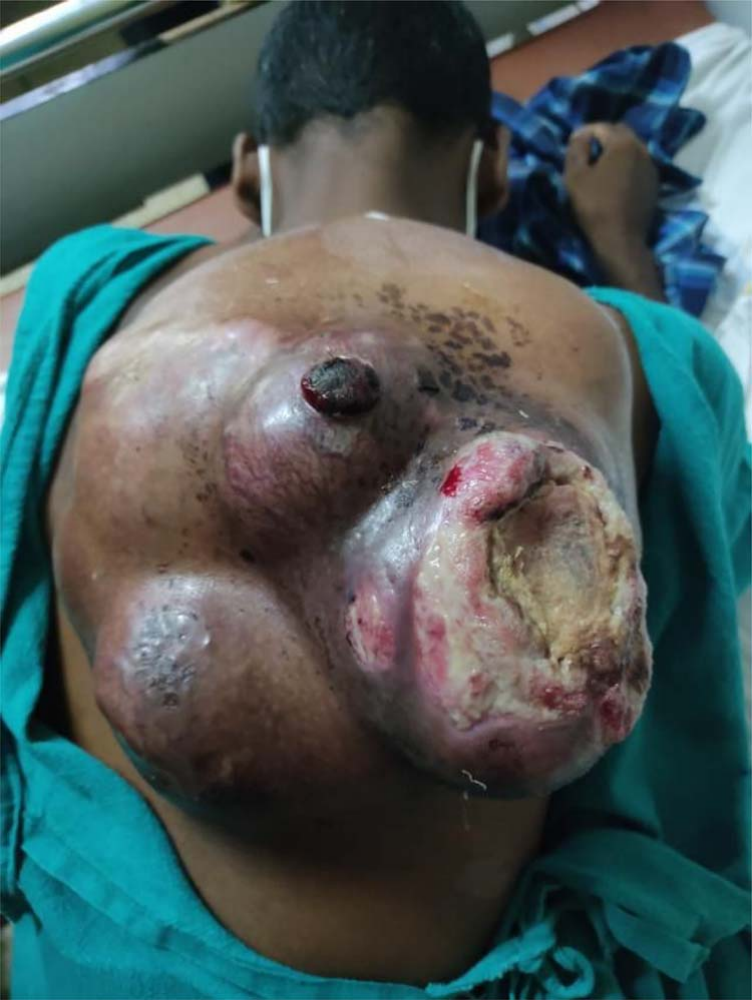
At the time of presentation. The picture shows a mass in the left upper back of size 20 × 20 cm^2^ with overlying ulceration and minimal discharge at the time of presentation.

**Figure 2:**
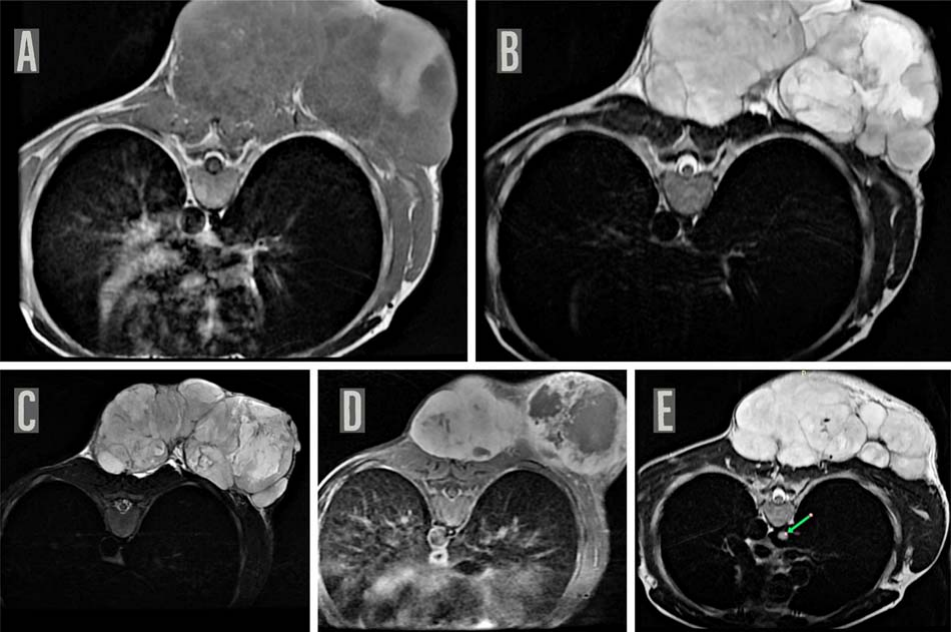
MRI chest. MRI of the chest on axial sections reveal a large well defined lobulated soft-tissue mass in the subcutaneous plane of the posterior chest wall, predominantly iso-hypointense on T1WI (**A**) with few areas of T1 hyperintensity and hyperintense on T2W image (**B**) and STIR images (**C**) and showing heterogenous enhancement with few central non-enhancing areas of necrosis on post contrast T1W images (**D**). Multiple internal septations were noted within the lesion with significant external contour bulge abutting the posterior chest wall muscles as well as bilateral posterior paraspinal muscles suggestive of a neoplastic process, possibly a mesenchymal tumor. MRI chest also shows a suspicious nodule (8 mm maximum diameter) appearing hyperintense on T2W image in the azygous lobe of right lung (indicated by green arrow) (**E**).

On local examination, he had a 20 × 20 cm^2^ tender hard mass at left upper back with overlying ulceration and discharge (Fig. 1). Magnetic resonance imaging (MRI) of the chest with contrast revealed a large lobulated heterogeneously enhancing soft-tissue mass in the subcutaneous plane of the posterior aspect of the chest wall along with a suspicious nodule in the azygous lobe of the right lung, which warranted further investigation (Fig. 2). Biopsy of the mass reported spindle cell sarcoma with possibility of neuronal cell origin. Immunophenotyping was advised for further confirmation but could not be done due to lack of resources.

**Figure 3:**
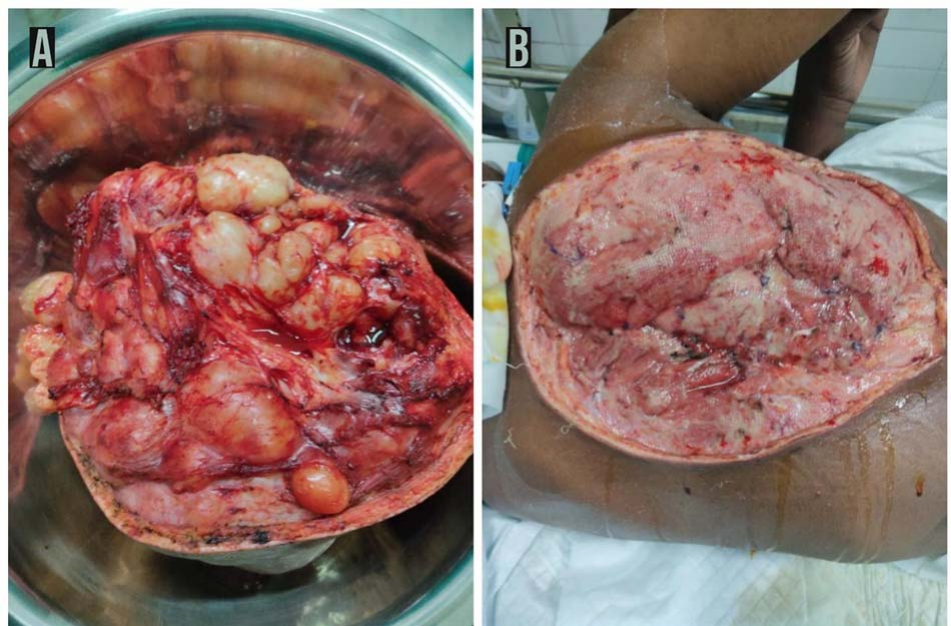
Post-operative findings. Post-operative picture of the excised part of the tumor showing 15 × 11 cm^2^ lobulated mass containing solid and cystic component with areas of necrosis and necrotic fluid with encapsulated well vascularized adhering to the surrounding structure extending deep up to ligamentum flavum but no encasing it (**A**). Post-operative picture of the posterior thorax showing the tumor base after wide en-block excision of the tumor (**B**).

The tumor was surgically excised by a team of surgeons (Fig. 3). The patient has been sent for radiotherapy of the tumor bed and further evaluation of his pulmonary nodule to the cancer hospital nearly 300-km away. Due to the unavailability of the plastic surgeon for skin grafting, the patient was again referred to another hospital nearly 150-km away.

## DISCUSSION

The evaluation of a patient with a suspected soft-tissue sarcoma includes history, imaging and biopsy [6]. MRI is the preferred modality for the evaluation of soft-tissue masses of the extremities, trunk, head and neck [7]. All patients diagnosed with sarcoma should also have a computed tomography scan of the chest at the time of diagnosis and in follow-up [6].

Spindle cell sarcomas affect people of almost any age and sex [8]. Two separate studies by Feng *et al*. and Smith *et al.* showed the median age at presentation of 57 years [9, 10]. In contrary to these studies, the age at presentation in our case is quite early (37 years). In the same study by Smith *et al*., the median tumor size found was 9.87 cm [10]. Our patient had a tumor size of 5 × 4 cm^2^ at the first presentation while it grew into a massive size of 20 × 20 cm^2^ when it recurred later after the surgery. This sort of presentation is quite rare as patients generally tend to present earlier with the mass with a median duration of 20 weeks [10], whereas our patient completely ignored the mass until it grew to a massive size and started hindering his day-to-day activities. This highlights that in poverty and destitution, people ignore medical attention until the very last stage [11].

Due to their rarity, delay or misdiagnosis is common for sarcomas especially in settings with limited facilities. [12, 13]. In our case, the mass was initially identified as liposarcoma. Later, repeat biopsies revealed spindle cell sarcoma. The rarity of the case and the little experience in diagnosing rare cases might have led to this error in histological diagnosis [14].

Two separate studies by Swamsura *et al.* and Diageler *et al.* reported local recurrence after surgery within a median duration of 19 months and 15.7 months, respectively [15, 16]. Our patient had a recurrence within 3–4 months after surgery, which is quite rare. A frequent follow-up is therefore advised after excision of sarcoma especially in the first 2 years [17].

In our case, the lack of proper communication between involved hospitals and doctors might be one of the reasons for his loss to follow-up [18, 19]. Besides his financial constraints and ignorance, inadequate counselling about the diagnosis may have led to the discontinuity of care, which is very common in developing countries [18, 19]. Due to limited resources in hospitals, it is common in developing countries to refer patients to other centers for further care [20]. Even tertiary care center like ours cannot provide comprehensive care to the patient. This creates a disadvantage in continuity-of-care for many patients. Hence, one of the main focuses regarding this patient would be to ensure adequate follow-up. To avoid such circumstances in the future, it is better if the health care team takes charge and arranges continuity-of-care for the patient so that any future patients do not leave without completing their course of treatment. Use of telemedicine is proven to be highly effective to ensure follow-up in developing countries [21]. It is, of course, necessary to address the main underlying issues like the financial burden for the patient and aim for sustainable treatment strategies.

## CONCLUSION

The management of soft-tissue sarcomas is best done in a center with appropriate expertise in multiple fields. In absence of appropriate expertise or resources, patients do not get adequate treatments in time. This may lead to grave consequences like metastasis or recurrence causing significant problems to the patients. Regular follow-up after treatment with history, physical examination and chest imaging is of utmost importance.

## CONFLICT OF INTEREST STATEMENT

None declared.

## FUNDING

None.
